# Mild hypothermia reduces endoplasmic reticulum stress‐induced apoptosis and improves neuronal functions after severe traumatic brain injury

**DOI:** 10.1002/brb3.1248

**Published:** 2019-03-04

**Authors:** Chuan‐Fang Wang, Cheng‐Cheng Zhao, Yi He, Zong‐Yang Li, Wen‐Lan Liu, Xian‐Jian Huang, Yue‐Fei Deng, Wei‐Ping Li

**Affiliations:** ^1^ Zhongshan School of Medicine Sun Yat‐sen University Guangzhou Guangdong China; ^2^ Brain Center Shenzhen Key Laboratory of Neurosurgery The First Affiliated Hospital of Shenzhen University Shenzhen Second People's Hospital Shenzhen Guangdong China; ^3^ Department of Neurosurgery Sun Yat‐sen Memorial Hospital Sun Yat‐sen University Guangzhou Guangdong China

**Keywords:** apoptosis, C/EBP‐homologous protein, endoplasmic reticulum stress, hypothermia, traumatic brain injury

## Abstract

**Background:**

Mild hypothermia is wildly used in clinical treatment of traumatic brain injury (TBI). However, the effect of mild hypothermia on endoplasmic reticulum (ER) stress‐induced apoptosis after severe TBI is still unknown.

**Methods:**

In the present study, we used BALB/c mice to investigate the efficacy of posttraumatic mild hypothermia in reducing ER stress. Severe TBI was induced by controlled cortical impact injury. Mild hypothermia treatment was performed immediately after surgery and maintained for 4 hr. The animals were euthanized at 1 and 7 days after severe TBI. The expression levels of ER stress marker proteins were evaluated using Western blot and immunofluorescence. Cell apoptosis rate was analyzed by TUNEL staining. Neuronal functions of the mice were assessed using rotarod test and Morris water maze.

**Results:**

Our results revealed that mild hypothermia significantly attenuated ER stress marker proteins, including p‐eIF2α/eIF2α, ATF4, CHOP and IRE‐1α, and reduced apoptosis rate in the pericontusion region at 1 and 7 days after severe TBI. Interestingly, mild hypothermia also prevented the translocation of CHOP into nucleus. In addition, posttraumatic mild hypothermia significantly improved neuronal functions after severe TBI.

**Conclusions:**

Our findings illustrated that mild hypothermia could reduce ER stress‐induced apoptosis and improve neuronal functions after severe traumatic brain injury.

## 1 INTRODUCTION

Traumatic brain injury (TBI) is a major cause of mortality and long‐term neurological disorder (Langlois, Rutland‐Brown, & Wald, 2006). It is estimated that between 18 and 250 per 100,000 persons suffer from TBI throughout the world annually (Popescu, Anghelescu, Daia, & Onose, 2015). TBI not only induces a primary, direct mechanical injury to the brain tissue, but also initiates a secondary, delayed progressive brain damage including extensive cell death and degeneration (Carbonell & Grady, 1999; Stoica & Faden, 2010).

The endoplasmic reticulum (ER) is an important cellular organelle that monitors protein synthesis, folding and modification, and also regulates Ca^2+^ homeostasis (Kim, Xu, & Reed, 2008; Larner, Hayes, & Wang, 2006). Pathologic insults, including TBI, cerebral ischemia and Alzheimer's disease, disturb normal ER function and lead to an accumulation of misfolded and unfolded proteins in the ER, which causes ER stress and unfolded protein response (UPR) (Halliday & Mallucci, 2014; Larner et al., 2006; Nakagawa et al., 2000; Xin et al., 2014). The UPR transiently attenuates protein translation and degrades of misfolded and unfolded proteins in the ER, which may lead to cell survival (Walter & Ron, 2011). However, prolonged and unresolved ER stress can trigger apoptotic cell death via activation of C/EBP‐homologous protein (CHOP) and inositol requiring kinase 1 (IRE‐1α) (Tabas & Ron, 2011). ER stress and UPR play a critical role in the secondary injury after TBI (Larner et al., 2006).

Mild hypothermia (33–35°C) is a potent neuroprotective therapy that is widely used in TBI treatment. To date, multiple cooling methods are available including cooling blankets and invasive intravascular cooling devices, which are widely used in the clinical setting (Holzer, 2008; Jiang, Yu, & Zhu, 2000). In experiment, mild hypothermia is usually induced by applying ice packs and ice blankets around the animals or administration of drugs (e.g. HPI‐201) (Gong et al., 2015; Lee et al., 2014; Lyeth, Jiang, & Liu, 1993). It has been proved that mild hypothermia can significantly reduce patients’ mortality rate and improve prognosis after severe TBI (Jiang et al., 2000; Jiang & Yang, 2007). Several researches also showed that mild hypothermia reduced intrinsic and extrinsic apoptotic pathways and prevented brain tissue from a secondary injury after TBI (Chen et al., 2016; Eroglu, Deniz, Kisa, Atasoy, & Aydinuraz, 2017; Zhang et al., 2017). However, the effect of mild hypothermia on ER stress‐induced apoptosis after severe TBI and the underlying mechanisms is still unknown.

In the present study, we used a mouse controlled cortical impact (CCI) injury model to investigate whether mild hypothermia reduces ER stress‐induced apoptosis and subsequently improves neuronal functions after severe TBI. The CCI injury model was produced by a controlled electrically driven impactor striking the left parieto‐temporal cortex of the anesthetized mice. After CCI, morphologic and cerebrovascular injury responses were commonly observed in experimental animals along with sustained sensory/motor and cognitive deficits (Fox, Fan, Levasseur, & Faden, 1998; Osier & Dixon, 2016; Scheff, Baldwin, Brown, & Kraemer, 1997).

## 2 METHODS

### 2.1 Animals

Adult (8‐ to 10‐week‐old) male BALB/c mice were housed for 7 days prior to surgery in the animal facility with controlled temperature and humidity and under standard 12‐hr light/dark cycles. Water and chow were provided ad libitum, and chow was withheld overnight prior to surgery. The animals were randomly divided into four groups: the sham injury with normothermia group (SNG; *n* = 25), the sham injury with hypothermia group (SHG; *n* = 25), the traumatic brain injury with normothermia group (TNG; *n* = 25), and the traumatic brain injury with hypothermia group (THG; *n* = 25). All the animal experiments were approved by the Institutional Animal Care and Use Committee of Sun Yat‐sen University, and all experiments were performed in accordance with the National Institute of Health Guide for the Care and Use of Laboratory Animals.

### 2.2 Surgical preparation, controlled cortical impact injury and temperature manipulation

The animal model of CCI injury and temperature manipulation were performed as previous described (Wang et al., 2016). Briefly, the mice were anesthetized with isoflurane and fixed in a stereotactic frame (Stoelting, Varese, Italy) prior to TBI. The body temperature of the mice was maintained at 37°C using a thermal heating blanket. Following a midline incision, a 4‐mm diameter circular craniotomy was made on the left side at a central location midway between the central incision and the temporalis muscle and midway between the bregma and lambda. During the procedure, the underlying dura should be kept intact. Prior to CCI, the impacting piston was adjusted at a 15°‐ to 20° angle so that the impacting tip (3‐mm diameter) was perpendicular to the exposed cortex. Then, the mice were subjected to a severe CCI injury using an electromagnetically controlled impacting device (PinPoint^™^ PCI3000 Precision Cortical Impactor^™^, Hatteras Instruments, Cary, NC). To achieve a severe brain injury, the deformation depths were set at 1.5 mm, and the piston velocity was set at 3.0 m/s. In the sham group, the mice received craniotomy without CCI injury.

The temperature manipulation was performed immediately after surgery. The brain and body temperatures were measured using temporalis muscle and rectal temperature probes, respectively. The brain temperatures of SHG and THG mice were maintained at 33°C, and those of SNG and TNG mice were maintained at 37°C. Hypothermia was achieved by placing the packed mice on ice packs and the mice were protected from direct contact with the ice packs. The target temperature was achieved within 30 min after surgery and maintained for 4 hr; SHG and THG mice were then slowly rewarmed to 37°C over 90 min.

### 2.3 Western blot

At 1 and 7 days after CCI, five mice from each group were anesthetized with sodium pentobarbital (100 mg/kg, i.p.) and transcardially perfused with 0.9% saline. Brain samples around the contusion region in the TBI group and those around the parietal craniotomy in the sham group were dissected and lysed in RIPA buffer system (Santa Cruz, California). Protein concentrations were measured using a BCA protein assay kit (Beyotime, Jiangsu, China). Equal amounts of protein for each sample were diluted in 5 × SDS loading buffer and denatured at 100°C for 5 min. Protein samples were separated by a 10% SDS‐PAGE gel or 15% SDS‐PAGE gel and transferred onto a 0.22 μm polyvinylidene difluoride membrane (Millipore, Merck KGaA, Darmstadt, Germany). Membranes were incubated overnight at 4°C with primary antibodies: rabbit anti‐ eIF2α (1:1000, Santa Cruz Biotechnology, Texas), rabbit anti‐p‐eIF2α (1:1000, Cell Signaling Technology, Beverly, MA), rabbit anti‐ATF4 (1:1000, Abcam, Cambridge, UK), rabbit anti‐CHOP (1:5000, Abcam, Cambridge, UK), rabbit anti‐IRE‐1α (1:1000, Cell Signaling Technology) and mouse anti‐GAPDH (1:10000, Bioworld Technology, MN). After washing, membranes were incubated for 1 hr at room temperature with the following secondary antibodies: goat anti‐mouse IgG‐HRP (1:5000, Bioworld Technology) and goat anti‐rabbit IgG‐HRP (1:5000, Bioworld Technology). The signals were detected using the ChemiDoc^™^ MP imaging system (Bio‐Rad Laboratories, Cressier, Switzerland).

### 2.4 Immunofluorescence

After survival periods of 1 and 7 days, five mice from each group were anesthetized with sodium pentobarbital (100 mg/kg, i.p.) and transcardially perfused with 0.9% saline, followed by fixation with 4% paraformaldehyde. Brain samples were removed and postfixed in 4% paraformaldehyde overnight at 4°C, followed by cryoprotection with 15%, 20%, 30% gradient sucrose solution for 48 hr at 4°C, respectively. Serial coronal sections (25 μm) were prepared using a freezing microtome. Before immunofluorescence staining, sections were rinsed three times with phosphate buffered saline (PBS) for 10 min and blocked for 2 hr with 10% goat serum in PBS containing 0.1% Triton X‐100. Sections were incubated overnight at 4°C with primary antibodies: rabbit anti‐CHOP (1:200, Abcam, Cambridge, UK), rabbit anti‐IRE‐1α (1:200, Cell Signaling Technology) and mouse anti‐NEUN (1:400, Abcam, Cambridge, UK). After primary antibody incubation, sections were rinsed three times in PBS for 10 min, followed by incubation with the appropriate Alexa Fluor‐tagged secondary antibody. DAPI was used for counterstaining. Sections were imaged using a Carl Zeiss confocal laser microscopy.

### 2.5 TUNEL staining

Cell apoptosis rate was assessed using a terminal deoxynucleotidyl transferase‐mediated dUTP nick‐end labelling (TUNEL) detection kit (Click‐iT^™^ plus TUNEL assay, Alexa Fluor^™^ 594, Thermo Fisher Scientific, Waltham, MA). According to the manufacturer's instructions, brain slices were incubated with TdT reaction mixture for 60 min at 37°C and then incubated with TUNEL reaction cocktail for 30 min at 37°C. DAPI was used for counterstaining. The TUNEL‐positive cell number and total cell number were counted and analyzed.

### 2.6 Rotarod test

The rotarod test was used for motor function analysis. The animals underwent three trials each day at an interval of 20 min. The test was performed at 1 day before surgery and 1 day, 7 days, and 14 days after surgery. During the test, a mouse was first placed on the rotarod for 10 s at 0 rpm. Then, the rotarod was gradually accelerated from 0 to 30 rpm and the speed was increased by 3 rpm in 10‐s intervals. The average latency to fall from the rotarod was recorded and analyzed for each group.

### 2.7 Morris Water Maze

Two weeks after CCI, spatial learning and memory functions were assessed using the Morris water maze. The maze was composed of a black circular tank (diameter 120 cm, depth 50 cm) and a black platform (diameter 6 cm, depth 30 cm) in the southwest quadrant of the tank. The tank was filled with water at 20 ± 1°C, and the platform was submerged 1 cm below the water surface. The mice received four trials per day for 5 days. In four different trials, the mice were released from one of the four quadrants (east, south, west and north), and allowed to swim until they found the platform, or for a maximum of 60 s. Then a mouse was allowed to stay on the platform for 20 s to build memories, no matter whether it found the platform or not. The latency to reach platform and mouse movement were recorded to analyze spatial learning and memory functions using a video tracking system (DigBehv, Jiliang Software Technology Company, Shanghai, China). On the sixth day, a probe test was performed, in which the platform in the southwest quadrant was removed. During the probe test, the mice were allowed to explore in the water pool for 60 s. After one probe trial, the mice were removed from the pool and all tracks were recorded using the video tracking system. The tracks were analyzed for the following parameters: the time spent in each quadrant and the distance traveled in each quadrant.

### 2.8 Statistical analysis

Statistical analysis was performed using SPSS 16.0. All data are presented as the means ± SEM. Homogeneity of the variances was tested with Levene Test. Statistical significance between experimental groups was determined using a one‐way analysis of variance (ANOVA) followed by Tukey's post‐hoc test when the homogeneity requirement was met (Sig. > 0.05) and one‐way Welch‐ANOVA followed by Games‐Howell post‐hoc test when it was not met (Sig. < 0.05). Comparison of neurons with nucleus translocation of CHOP between the TNG and THG were performed using Student's *t*‐test. A value of *p *<* *0.05 was considered statistically significant.

## 3 RESULTS

### 3.1 Mild hypothermia reduced ER stress after severe TBI

**Figure 1:**
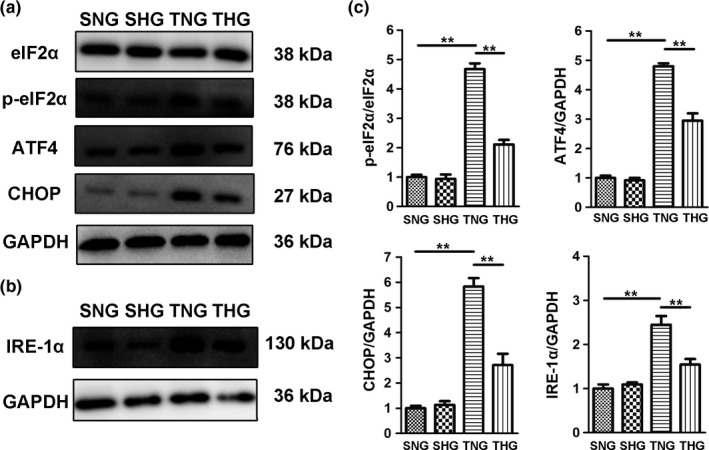
Mild hypothermia treatment reduced the expression of ER stress marker proteins p‐eIF2α/eIF2α, ATF4, CHOP and IRE‐1α in the pericontusion region at 1 day after severe TBI. (a) Representative immunoblots of p‐eIF2α, eIF2α, ATF4 and CHOP protein expression in the pericontusion region of the SNG, SHG, TNG and THG at 1 day after severe TBI. GAPDH served as a loading control. (b) Representative immunoblots of IRE‐1α protein expression in the pericontusion region of the SNG, SHG, TNG and THG at 1 day after severe TBI. GAPDH served as a loading control. (c) Quantitative analyses of p‐eIF2α/eIF2α, ATF4, CHOP and IRE‐1α expression levels were performed. The results of these analyses were normalized to GAPDH level. The data are represented as means ± *SEM*,* n* = 5, **p <* *0.01, TNG versus THG or sham. SNG, the sham injury with normothermia group; SHG, the sham injury with hypothermia group; TNG, the traumatic brain injury with normothermia group; THG, the traumatic brain injury with hypothermia group

**Figure 2:**
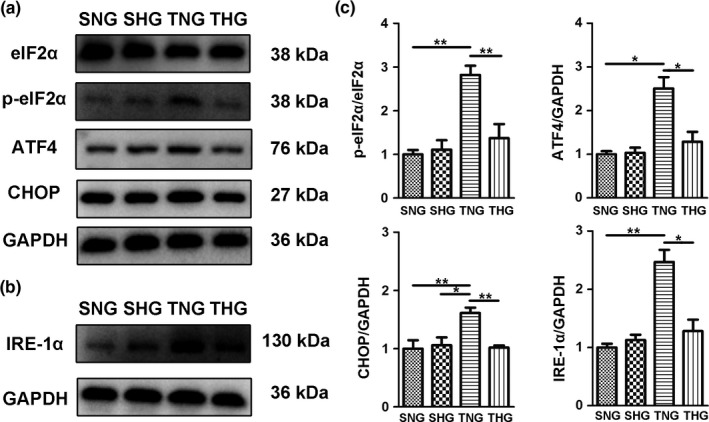
Mild hypothermia treatment reduced the expression of ER stress marker proteins p‐eIF2α/eIF2α, ATF4, CHOP and IRE‐1α in the pericontusion region at 7 days after severe TBI. (a) Representative immunoblots of p‐eIF2α, eIF2α, ATF4 and CHOP protein expression in the pericontusion region of the SNG, SHG, TNG and THG at 7 days after severe TBI. GAPDH served as a loading control. (b) Representative immunoblots of IRE‐1α protein expression in the pericontusion region of the SNG, SHG, TNG and THG at 7 days after severe TBI. GAPDH served as a loading control. (c) Quantitative analyses of p‐eIF2α/eIF2α, ATF4, CHOP and IRE‐1α expression levels were performed. The results of these analyses were normalized to GAPDH level. The data are represented as means ± *SEM*,* n* = 5, *p <* *0.05, **p <* *0.01, TNG versus THG or sham. SNG, the sham injury with normothermia group; SHG, the sham injury with hypothermia group; TNG, the traumatic brain injury with normothermia group; THG, the traumatic brain injury with hypothermia group

Western blot was performed to evaluate the expression levels of ER stress‐associated proteins at 1 and 7 days after severe TBI. As shown in Figure 1 and Figure 2, the expression levels of p‐eIF2α/eIF2α, ATF4, CHOP and IRE‐1α were dramatically increased at 1 (Figure 1) and 7 days (Figure 2) after severe TBI compared with the SNG and SHG. Posttraumatic mild hypothermia treatment significantly reduced the expression levels of these proteins. Moreover, the expression levels of p‐eIF2α/eIF2α, ATF4, CHOP and IRE‐1α dropped to normal levels in THG at 7 days after severe TBI.

**Figure 3:**
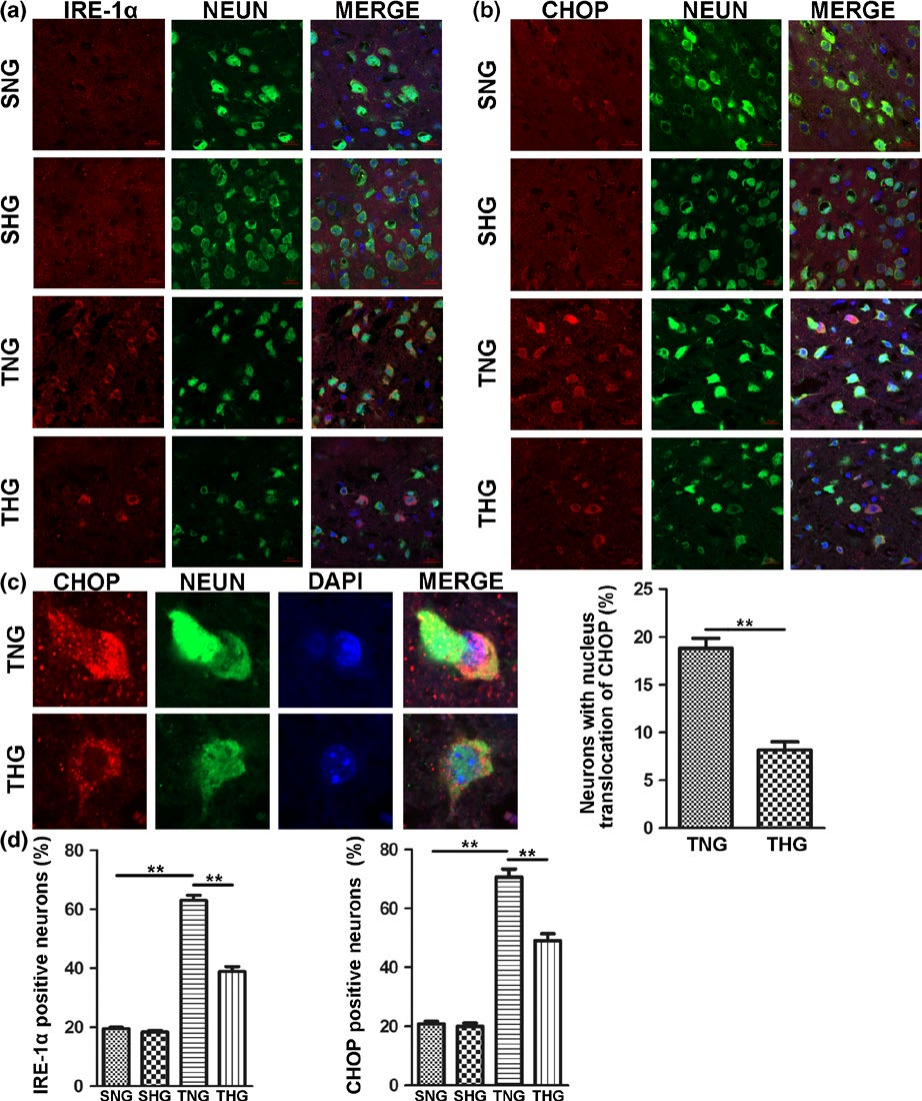
Effect of mild hypothermia on expression of CHOP and IRE‐1α in neurons after severe TBI. (a) Confocal microscopic images of IRE‐1α and NEUN expression in the pericontusion region at 1 day after severe TBI. (b) Confocal microscopic images of CHOP and NEUN expression in the pericontusion region at 1 day after severe TBI. (c) DAPI, CHOP and NEUN co‐staining revealed that CHOP translocated into nucleus in neurons after severe TBI and posttraumatic hypothermia could impede this process. (d) Quantification of the number of IRE‐1α positive neurons and CHOP positive neurons. The data are represented as means ± *SEM*,* n* = 5, **p <* *0.01, TNG versus THG or sham. SNG, the sham injury with normothermia group; SHG, the sham injury with hypothermia group; TNG, the traumatic brain injury with normothermia group; THG, the traumatic brain injury with hypothermia group

CHOP and IRE‐1α expression in neurons was further assessed by immunofluorescence staining with NEUN at 1 day after TBI. As shown in Figure 3, CHOP and IRE‐1α were mainly colocalized with NEUN in the pericontusion region at 1 day after TBI. Consistent with the results of Western blot, CHOP and IRE‐1α positive neurons was significantly increased at 1 day after TBI compared with the SNG and SHG (*p *<* *0.01, Figure 3d). Moreover, mild hypothermia dramatically decreased CHOP and IRE‐1α positive neurons after severe TBI (*p *<* *0.01, Figure 3d). Interestingly, we found CHOP translocated into nucleus in many neurons after severe TBI, which was barely seen in the SNG and SHG. However, posttraumatic hypothermia could significantly decreased the number of neurons with nucleus translocation of CHOP (*p *<* *0.01, Figure 3c).

### 3.2 Mild hypothermia reduced apoptosis after severe TBI

**Figure 4:**
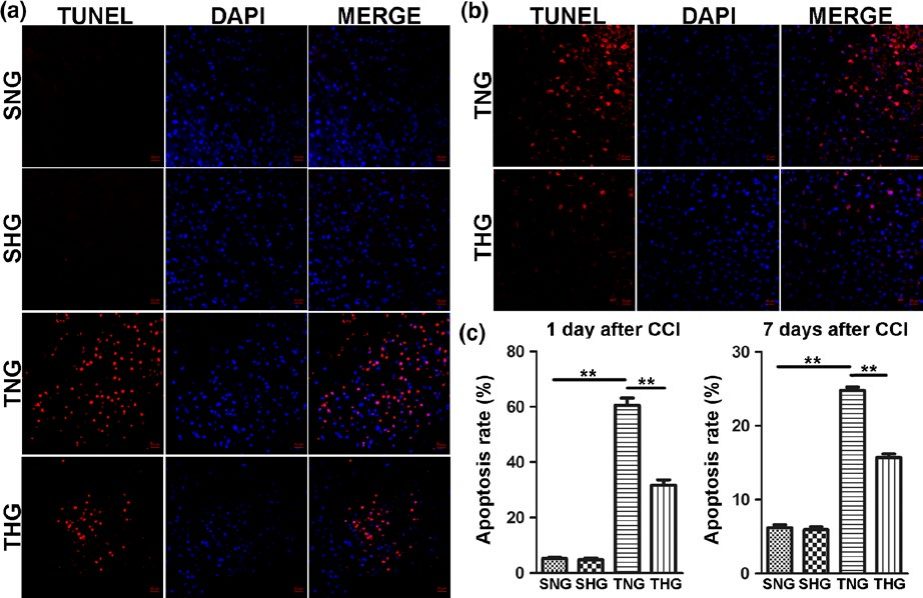
Mild hypothermia reduced cell apoptosis rate at 1 and 7 days after severe TBI. (a) Apoptotic cells detected by TUNEL staining in the pericontusion region at 1 day after severe TBI. (b) Apoptotic cells detected by TUNEL staining in the pericontusion region at 7 days after severe TBI. (c) Quantitative analyses of cell apoptosis rate were performed. The data are represented as means ± *SEM*,* n* = 5, **p <* *0.01, TNG versus THG or sham. SNG, the sham injury with normothermia group; SHG, the sham injury with hypothermia group; TNG, the traumatic brain injury with normothermia group; THG, the traumatic brain injury with hypothermia group

TUNEL staining was performed to assess the apoptosis rate in the pericontusion region at 1 and 7 days after CCI. As shown in Figure 4, the apoptosis rate was significantly increased in the TNG and THG, whereas apoptotic cells were barely detected in the SNG and SHG. Moreover, mild hypothermia significantly reduced cell apoptosis in the pericontusion region compared with the TNG at 1 and 7 days after severe TBI (*p *<* *0.01, Figure 4).

### 3.3 Mild hypothermia improved neuronal functions after severe TBI

**Figure 5:**
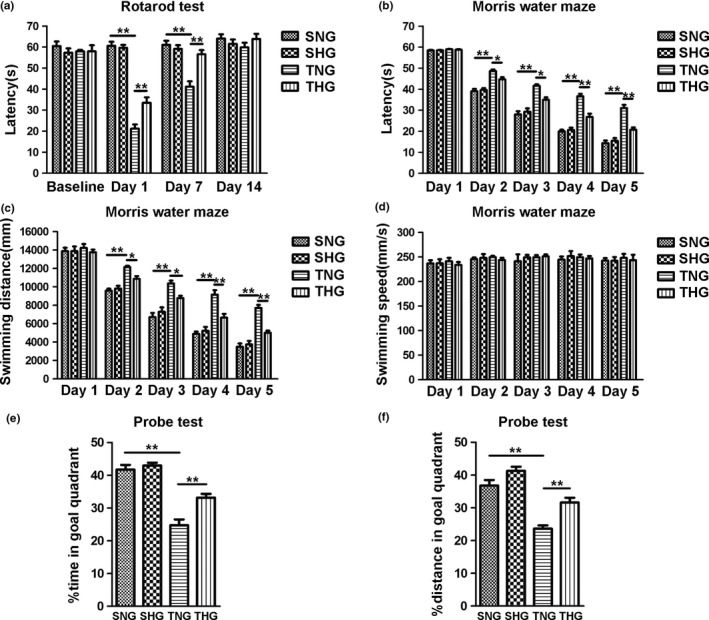
Mild hypothermia treatment improved neuronal functions in mice after severe TBI. (a) Quantitative analyses of the average latency to fall from the rotarod were performed. (b, c, d) Quantitative analyses of (b) the average latency, (c) swimming distance and (d) swimming speed were performed. (e, f) Probe test of the Morris Water Maze. Quantitative analyses of (e) the percentage of time spent in the goal quadrant (southwest quadrant) and (f) the percentage of distance traveled in the goal quadrant were performed. The data are represented as means ± *SEM*,* n* = 5, *p <* *0.05, **p <* *0.01, TNG versus THG or sham. SNG, the sham injury with normothermia group; SHG, the sham injury with hypothermia group; TNG, the traumatic brain injury with normothermia group; THG, the traumatic brain injury with hypothermia group

We performed the rotarod test to assess the effect of mild hypothermia on motor function after severe TBI. The average latency to fall from the rotarod was analyzed for each group. As Figure 5a shows, latency in all groups had no significant difference at 1 day before surgery. After severe TBI, the latency of the TNG was dramatically decreased compared to the SNG and SHG at 1 and 7 days after surgery (*p *<* *0.01, Figure 5a). However, posttraumatic mild hypothermia treatment significantly attenuated the dropping of latency after severe TBI at these two time points. Meanwhile, the latency of the THG had no significant difference compared to the SNG and SHG at 7 days after surgery. Fourteen days after surgery, latency in all groups had no significant difference. These results revealed that mild hypothermia improved motor function of the mice after severe TBI.

The Morris Water Maze test was also performed to assess the learning and memory functions of the mice after severe TBI. In the acquisition training (day 1–5), latency, swimming speed and swimming distance were analyzed for each group. As shown in Figure 5b,c and d, latency and swimming distance in all groups had no significant difference on the first experiment day. Mice in the TNG spent more time and swam more distance finding the platform since the second experiment day. On the fifth day, the average latency and swimming distance of the TNG were still more than twofold greater than the SNG and SHG. However, mild hypothermia significantly decreased the latency and swimming distance after severe TBI since the second day. The swimming speed in all groups showed no significant difference since the first day. In the probe test (day 6), the percentage of time spent and the percentage of distance traveled in the goal quadrant (southwest quadrant) were analyzed for each group. As shown in Figure 5e and f, mice in the TNG spent significantly less time and traveled a significantly smaller portion of their path in the goal quadrant than mice in the THG. These results revealed that mild hypothermia improved learning and memory functions of the mice after severe TBI.

## 4 DISCUSSION

Many studies have shown that posttraumatic ER stress plays a critical role in the secondary injury after TBI (Begum et al., 2014; Larner et al., 2006). In the current study, we detected persistent activation of the IRE‐1α and PERK pathways at both 1 and 7 days after severe CCI. Such prolonged activation of IRE‐1α and PERK‐ATF4‐CHOP can induce cell apoptosis and cause secondary injury. Posttraumatic hypothermia has been proved to be an efficient method to prevent intrinsic and extrinsic apoptotic pathways after TBI. We found therapeutic hypothermia could also significantly decrease the expression level of ER stress marker proteins including p‐eIF2α/eIF2α, ATF4, CHOP and IRE‐1α compared with TNG at both 1 and 7 days after severe TBI. Furthermore, therapeutic hypothermia could reduce cell apoptosis and improve neuronal functions after severe TBI. Our findings suggest that the neuroprotective effects of therapeutic hypothermia might be exerted partially through suppression of prolonged ER stress‐induced apoptosis.

ER stress activates three principal stress sensor pathways, IRE‐1, PERK and ATF6 pathways (Ron & Walter, 2007). These pathways constitute an ER‐specific UPR which can ameliorate the accumulation of unfolded proteins and maintain homeostasis in the ER (Walter & Ron, 2011). However, prolonged activation of IRE‐1α and CHOP can induce widespread cell apoptosis in various neurodegenerative disorders including TBI, cerebral ischemia and Alzheimer's disease (Kim et al., 2008; Larner et al., 2006; Szegezdi, Logue, Gorman, & Samali, 2006).

IRE‐1α is a transmembrane kinase and also an endoribonuclease. When activated, IRE‐1α can recruit the adaptor protein tumor necrosis factor receptor‐associated factor 2 (TRAF2) and finally activate its downstream target c‐Jun N‐terminal kinase 1 (JNK) (Urano et al., 2000). The JNK branch of IRE‐1α pathway regulates and activates cell apoptosis (Ventura et al., 2006). IRE‐1α also involves in the mitochondrial pathway of apoptosis through affecting Bak and Bax (Hetz et al., 2006). In the present study, our results revealed that mild hypothermia could dramatically decrease that expression of IRE‐1α after severe TBI.

PERK is an ER‐resident transmembrane kinase. When activated, PERK phosphorylates eIF2α and results in the expression of transcription factors ATF4 and CHOP. ATF4/CHOP axis plays a crucial role in ER stress‐induced cell apoptosis (Larner et al., 2006; Tabas & Ron, 2011). ATF4 is a transcriptional regulator that belongs to the cAMP response element‐binding protein 2 family (Vattem & Wek, 2004). Under conditions of ER stress, enhanced levels of ATF4 can migrate to nucleus and bind targeted DNAs to regulate their transcription including CHOP (Han et al., 2013). CHOP is a transcription factor that translocates into nucleus to control genes encoding components involved in cell apoptosis (Iurlaro & Munoz‐Pinedo, 2016). It has been proved that prolonged activation of CHOP suppresses the expression of the pro‐survival protein Bcl‐2 and activates pro‐apoptotic protein Bax (Fu et al., 2010; McCullough, Martindale, Klotz, Aw, & Holbrook, 2001). CHOP can also activate ER oxidoreductin 1α (ERO1α), an oxidizing enzyme that regulates reactive oxygen species (ROS). Increased ERO1α expression may promote a hyperoxidizing environment and lead to ROS‐induced cell apoptosis (Rao et al., 2015). In our study, we found that mild hypothermia could significantly decrease the expression of ATF4 and CHOP after severe TBI. Moreover, mild hypothermia prevented the translocation of CHOP into cell nucleus which might impede the transcription of cell apoptosis‐associated proteins.

In conclusion, we investigated the efficacy of therapeutic hypothermia in reducing ER stress and ER stress‐induced apoptosis after severe TBI. Severe TBI triggered sustained expression of PERK and IRE‐1α pathway marker proteins including p‐eIF2α/eIF2α, ATF4, CHOP and IRE‐1α at 1 and 7 days after TBI. Therapeutic hypothermia attenuated PERK and IRE‐1α pathway marker proteins expression and reduced prolonged ER stress‐induced apoptosis. Moreover, the THG mice exhibited a better recovery of motor, learning and memory functions after severe TBI. These findings revealed a new mechanism of therapeutic hypothermia on preventing cell apoptosis after severe TBI. Further researches are warranted to study the underlying mechanism of therapeutic hypothermia on regulating ER stress.

## DISCLOSURE

No competing financial interests exist.

## CONFLICT OF INTEREST

None declared.
